# Percutaneous Coronary Intervention vs. Coronary Artery Bypass Grafting in Left Main Coronary Artery Disease: An Updated Systematic Review and Meta-Analysis

**DOI:** 10.7759/cureus.48297

**Published:** 2023-11-05

**Authors:** Conor Hennessy, John Henry, Gokul Parameswaran, Devon Brameier, Rajesh Kharbanda, Saul Myerson

**Affiliations:** 1 Medicine and Surgery, Nuffield Department of Surgical Sciences, John Radcliffe Hospital, Oxford, GBR; 2 Medicine and Surgery, Oxford University Clinical Academic Graduate School, Oxford University Hospitals Foundation Trust, Oxford, GBR; 3 Medicine and Surgery, University of Oxford, Oxford, GBR; 4 Medicine and Surgery, Medical School Offices, Medical Sciences Division, University of Oxford, Oxford, GBR; 5 Cardiology, Oxford Heart Centre, National Institute for Health and Care Research (NIHR) Biomedical Research Centre, Oxford University Hospitals, Oxford, GBR; 6 Cardiology, Division of Cardiovascular Medicine, Radcliffe Department of Medicine, Oxford, GBR; 7 Cardiology, Department of Cardiology, Oxford University Hospitals NHS Foundation Trust, Oxford, GBR

**Keywords:** percutaneous coronary intervention, coronary artery bypass graft, lmca disease, survival mi, drug-eluting stents (des)

## Abstract

Recently, both US and European guidelines have predominantly recommended coronary artery bypass grafting (CABG) as the preferred revascularisation method. However, emerging data have raised the possibility of percutaneous coronary intervention (PCI) being a viable and effective alternative. This meta-analysis sought to evaluate the latest insights from major clinical trials to ascertain whether PCI could be as effective as CABG in treating left main coronary artery (LMCA) disease.

To achieve this, a comprehensive systematic search was conducted across databases, including Medline (via PubMed), Embase, Cochrane, and clinicaltrials.gov. The search spanned from the inception of these databases to August 20, 2022, and exclusively focused on randomized controlled trials (RCTs). Employing the random effects model, selected studies underwent rigorous analysis. The study outcomes encompassed a spectrum of factors such as all-cause mortality, major adverse cerebrovascular and cardiovascular events (MACCE), myocardial infarction (MI), stroke, and revascularisation procedures. The observation periods of interest included the 30-day mark, 1 year, 5 years, and 10 years.

The analysis integrated six RCTs, revealing noteworthy patterns. In terms of all-cause mortality, PCI demonstrated non-inferiority to CABG across all observed time frames: 30 days (OR 0.6), 1 year (OR 0.77), 5 years (OR 1.41), and 10 years (OR 1.08). Analysis of MACCE outcomes favored PCI at 30 days and CABG at 5 years. The utilisation of the original five-year EXCEL (Evaluation of XIENCE versus Coronary Artery Bypass Surgery for Effectiveness of Left Main Revascularisation) trial definition for MI highlighted higher MI rates for PCI compared to CABG (OR 1.66, P < 0.05). Intriguingly, when the subsequently released EXCEL data, aligned with the third universal MI definition, was incorporated, the five-year data consistently leaned towards CABG. Specifically, the PCI group exhibited 7.5% MI rates in contrast to the 3.6% in the CABG cohort (OR 2.19, P < 0.001). Concerning stroke, PCI proved advantageous at 30 days and 1 year while exhibiting no significant disparity at 5 and 10 years. Revascularisation procedures favoured CABG at one and five years, with comparability at the remaining time points.

In summation, the outcomes of this comprehensive meta-analysis suggest that PCI could serve as a feasible alternative to CABG in the context of uncomplicated LMCA disease. It's worth noting that CABG might still hold an advantage for complex lesions.

## Introduction and background

Left main coronary artery (LMCA) disease, defined as angiographic stenosis of >50% luminal diameter, poses a significant risk of excess mortality. Historically, medically managed LMCA disease has been associated with a three-year mortality rate of 40-50% although this data predates modern medical management [1]. The high mortality is attributed to the extensive area of myocardium supplied by the artery, accounting for up to 70% of the myocardium in right-dominant hearts and up to 100% in left-dominant hearts [2]. Additionally, extensive coronary atheroma may contribute to the poorer prognosis associated with LMCA occlusion when compared to other coronary arteries [3].

The optimal revascularisation approach for LMCA lesions has been a topic of debate over the past decades. The European and US guidelines have traditionally recommended coronary artery bypass grafting (CABG) as the standard of care for LMCA disease management [4]. However, recent studies have suggested that percutaneous coronary intervention (PCI) with drug-eluting stents (DES) may offer a low risk of mortality at intermediate and long-term follow-up after left main stenting [5,6]. As a result, recent European guidelines propose that PCI may be effective in treating anatomically simple LMCA lesions while still recommending CABG for more severe or anatomically complex diseases [1].

The changes in guidelines have been informed largely by six randomised controlled clinical trials comparing PCI with drug-eluting stents to CABG for the treatment of LMCA disease. However, the results of these studies have been conflicting; an issue further compounded by low participant numbers in some studies. Several meta-analyses have been performed aiming to determine the optimal intervention, but despite drawing from similar studies, the results remain inconclusive. This may be due to the heterogeneity of the studies included with many systematic reviews including both randomised control trials as well as retrospective observational studies in the same analysis [7-9]. Other reviews have excluded key trials or have included too few outcomes or follow-up time points in their analysis [10-12]. Another issue may be variation in surgical or interventional techniques such as the use of drug-eluting stents as well as more general technical or surgical developments over time. For example, the use of on- and off-pump in CABG varied significantly across studies, from 15% off-pump in the SYNTAX trial to 46% off-pump in the Bourdroit study, contributing to outcome variation.

One study that aimed to address some of the previous trial's limitations is the EXCEL (Evaluation of XIENCE versus Coronary Artery Bypass Surgery for Effectiveness of Left Main Revascularisation) trial. With almost two thousand participants, it nearly doubled the total number of participants in prior trials analysing PCI and CABG in LMCA disease. The trial's three-year and five-year data in 2016 and 2019 respectively, demonstrated non-inferiority of PCI when compared to CABG. Despite this, the trial faced criticism from cardiologists and cardiac surgeons, particularly concerning the definition of myocardial infarction (MI) used, which some believed unfairly favoured surgical revascularisation [13]. However, the EXCEL trial authors have responded to this criticism by providing additional MI data which showed they had adhered to the Third Universal Definition of MI [14].

Given recent data from the EXCEL trial as well as an additional 10-year follow-up from other trials, it is timely to conduct a meta-analysis exclusively comparing PCI and CABG for LMCA disease. By focusing solely on randomised controlled trials, this analysis aims to provide a comprehensive and unbiased comparison to inform clinical decision-making for LMCA disease management.

## Review

Materials and methods

The review protocol for this systematic review and meta-analysis was prospectively registered with PROSPERO (ID: CRD42021230350). Following the PRISMA (Preferred Reporting Items for Systematic Reviews and Meta-Analyses) guidelines, we meticulously constructed this study to investigate the optimal revascularisation approach for LMCA disease [15]. The databases utilised for this review were MEDLINE (via PubMed) and EMBASE, supplemented by a search of Clinicaltrials.gov. To formulate our research question, we employed the PICO-S method - Population (Patients with Left main coronary artery disease); Intervention (Percutaneous coronary intervention); Comparison (Coronary artery bypass grafting); Outcome (All-cause mortality, Myocardial infarction, stroke, major adverse cardiovascular/cerebrovascular events (MACCE), revascularisation); Study type (Randomised controlled trials). The comprehensive search strategy involved a combination of relevant concept terms, employing free text terms, synonyms, and controlled vocabulary terms (MeSH for MEDLINE and EMTREE for EMBASE). The search was conducted from January 4 to April 20, 2021, with an updated search in August 2022 revealing no relevant studies. The Cochrane Handbook for Systematic Reviews of Interventions guided the study selection and analysis [16]. Table 1 outlines the search terms used.

**Table 1:** Outline of search terms used The research question was split into 4 concepts and each concept was expanded to ensure all possible variations of the concept were explored. MeSH terms and EMTREE terms were also searched. LMCA: left main coronary artery; LCA: left coronary artery); PCI: percutaneous coronary angioplasty, DES: drug-eluting stent; CABG: coronary artery bypass graft

	Concept 1	Concept 2	Concept 3	Concept 4
Key concepts	Left main coronary artery disease	Percutaneous coronary intervention	Coronary artery bypass graft	All-cause mortality
Free-text terms / natural language terms (synonyms, UK/US terminology, medical/laymen’s terms, acronyms/abbreviations, drug brands, more narrow search terms)	Left main disease, LMCA disease, LCA disease, Left coronary artery disease, Left main stem disease, Left coronary artery, LCA	PCI, Drug-eluting stent, Balloon coronary angioplasty, DES, Percutaneous transluminal coronary angioplasty	CABG, Surgical revascularisation, Surgical revascularisation, Surgical revascularization	Death, Mortality, Morbidity
Controlled vocabulary terms / Subject terms (MeSH terms, Emtree terms)	Coronary artery disease, Left main coronary artery	Drug-eluting stents, Angioplasty, Percutaneous transluminal angioplasty	Bypass surgery, Coronary artery Bypass, Coronary artery	Determinant, Mortality Determinants, Mortality Morbidity

Two independent screeners conducted title and abstract screening, followed by full-text screening of articles, with disputes resolved by a third party. The inclusion criteria focused on randomised controlled trials (RCTs) comparing PCI and CABG for left main vessel disease, with a minimum follow-up of one year and reporting all-cause mortality as an outcome. Exclusion criteria comprised non-randomised trials, retrospective studies, articles without available full texts, and articles not in English.

The outcomes of interest for this review were all-cause mortality (death during the follow-up period by any means), stroke (any cerebrovascular event causing neurological impairment lasting over 24 hours in the period following the initial intervention), MI, revascularisation (any incidence of repeated revascularisation by any means following the initial intervention) and MACCE (combination of mortality, stroke, MI and repeat revascularisation). Definitions of MI varied between studies, and we analysed this outcome using both the original EXCEL definition and the Third Universal Definition of MI, considered more standard practice, as per the trial authors' recent re-analysis of five-year outcome data. All studies measured troponin levels as well as ECG for the diagnosis of MI.

Data collection encompassed study characteristics (e.g., authors, publication year, patient numbers, geographic localisation of participants) and outcome data (e.g., all-cause mortality, MACCE, and revascularisation) of each trial included in the analysis. Data was extracted as event numbers compared to the total population of each trial group as reported in the individual trials. Meta-analysis was conducted using Review Manager software (RevMan 5.2, Cochrane Collaboration, Nordic Cochrane Centre, Copenhagen, Denmark), as per the published protocol. Study event numbers were entered as dichotomous data into the program, with events relative to total participants used to calculate the odds ratio (OR) with a 95% confidence interval also calculated. P-value < 0.05 was considered significant. A random effects model was used, and the statistical method used was the Mantel-Haenszel method which was deemed appropriate due to the nature of the data (dichotomous i.e. event/non-event).

To assess bias, we used the Cochrane Handbook for Systematic Reviews and Meta-Analyses addressing domains were random sequence generation, allocation concealment, blinding of participants and personnel, blinding of outcome assessment, incomplete outcome data and selective reporting [16].

Results

**Figure 1:**
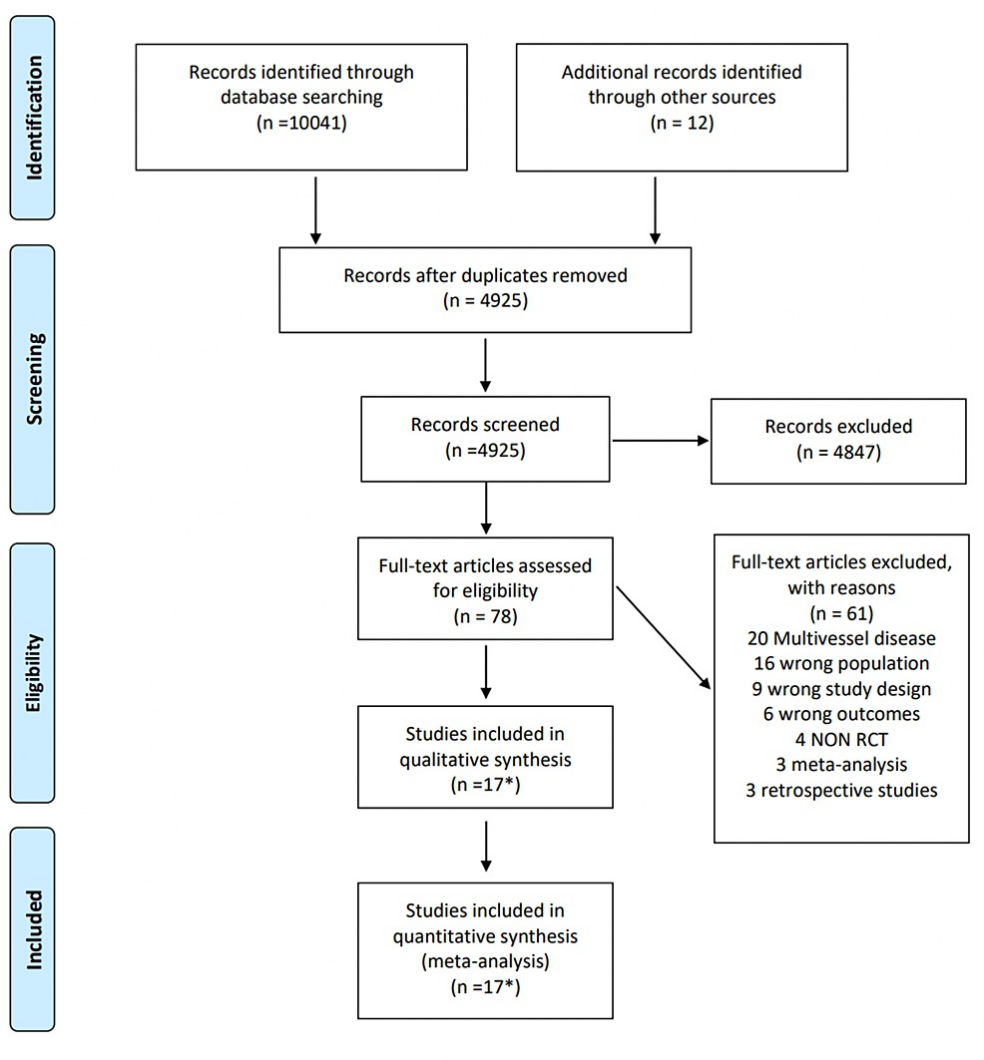
PRISMA flow diagram of study selection and screening The diagram outlines the number of citations produced by the selected search terms. * The 17 studies that were included in the meta-analysis described the outcomes of six unique RCTs. PRISMA: Preferred Reporting Items for Systematic Reviews and Meta-Analyses; RCT: randomized controlled trials

A total of 4545 articles were retrieved from the MEDLINE search, and an additional 5946 were retrieved by searching EMBASE. An additional 12 citations were retrieved from clinicaltrials.gov (Figure 1). After removing duplicates, 4925 studies proceeded to title and abstract screening. Following the exclusion of irrelevant studies and those not meeting the inclusion and exclusion criteria, 78 full texts were assessed for eligibility. Ultimately, 17 texts were included for final analysis, encompassing 6 randomised control trials published at different time points after follow-up (Table 2). All included studies focused on comparing PCI vs. CABG in LMCA disease. Notably, the Synergy between Percutaneous Coronary Intervention with Taxus and Cardiac Surgery (SYNTAX) study included was the LMCA sub-study of the larger SYNTAX trial, examining both LMCA disease and multivessel disease. Data from all included studies were published between 2008 and 2020, involving a total of 4700 participants across all trials, with 2349 patients in the PCI groups and 2594 participants in the CABG groups. Follow-up time points analysed for this study included 30 days, 1 year, 5 years, and 10 years post-intervention, with respective participant numbers of 3194, 2795, 4394 and 705.

**Table 2:** Study details PCI: percutaneous coronary intervention; CABG: coronary artery bypass graft; MACCE: major adverse cerebrovascular and cardiovascular events; LVEF: left ventricular ejection fraction; CC DP: cobalt-chromium drug-eluting platform; SS DP: stainless steel drug-eluting platform; IMA: internal mammary artery

Study	Patient No.	Follow-up	Countries	Primary outcome	Stent platform	% CABG grafts from IMA	% CABG use off-pump
EXCEL [17]	PCI: 958 CABG: 957	30 d, 3 years, 5 years	Asia, Australia, North America, South America, Europe	All-cause mortality, stroke or MI at 3 years	Everolimus eluting CC DP (2^nd^ Gen)	96.8% IMA	Off (29.4%) On (71.6%)
SYNTAX [18]	PCI: 357 CABG: 348	1 year, 2 years, 5 years	North America and Europe	MACCE score at 1 year, All-cause mortality, stroke, MI, revascularisation	Paclitaxel eluting SS DP (1^st^ gen)	NR	Off (15%) On (85%)
PRECOMBAT [19,20]	PCI: 300 CABG: 300	1 year, 5 years, 10 years	South Korea	MACCE for 12 months post-intervention	Sirolimus eluting SS DP (1^st^ gen)	100% IMA	Off (42%) On (58%)
NOBLE [21]	PCI: 592 CABG: 592	30 days, 1 year, 5 years	Nordic and Baltic countries and the UK	MACCE post intervention	12% sirolimus eluting SS DP, 88% biolimus eluting CC BP (2^nd^ gen)	86% L-IMA, 8% R-IMA, 6% Other	Off (16%) On (84%)
LE MANS [22]	PCI: 52 CABG: 53	30-day, 1 year, 10 years	North America	LVEF 1-year post-intervention	Bare metal (65%), Paclitaxel eluting SS DP (35%)	72% IMA	Off (1.9% n =1) On (98.1%)
Bourdroit 2011 [23]	PCI: 100 CABG: 101	30-day, 1 year	Germany	All-cause mortality, MI, revascularisation	Sirolimus eluting SS DP (1^st^ gen)	100% IMA (1 patient other)	Off (46%) On (54%)

**Figure 2:**
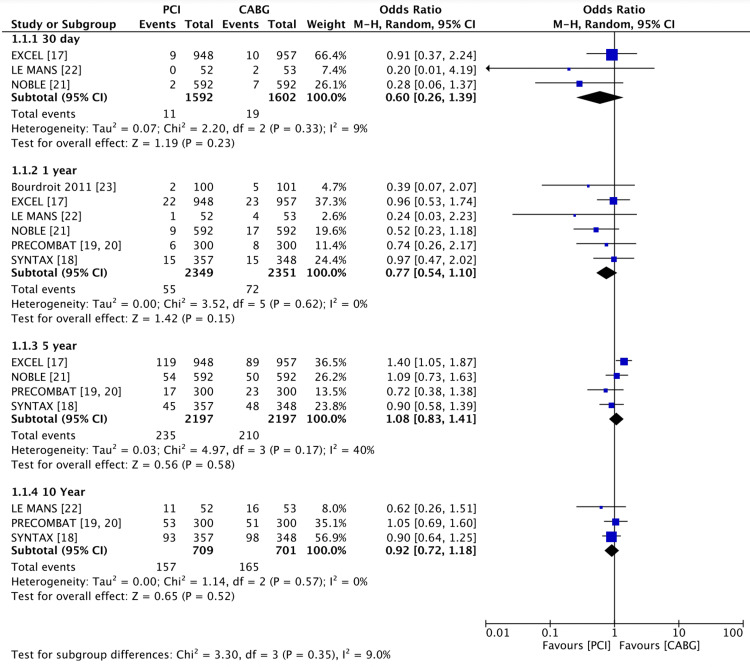
Forest plot of all-cause mortality PCI vs CABG Data shown compares the outcome of all-cause mortality in left main coronary artery disease when treated with PCI vs CABG [17-23]. PCI: percutaneous coronary intervention; CABG: coronary artery bypass grafting. P < 0.05 was considered significant.

Regarding all-cause mortality (Figure 2), there was no significant difference between the PCI group and the CABG group at 30 days follow-up (0.69% vs 1.2%, OR 0.6 (95% CI 0.26, 1.39), P = 0.23, I2 = 9%), 1-year follow-up (2.3% vs 3.1%, OR 0.77 (95% CI 0.54, 1.10), P = 0.15, I2 = 0%), 5 years follow- up (10.7% vs 10.5%, OR 1.08 (95% CI 0.83, 1.41), P = 0.58, I2 = 40%), or 10 years follow-up (22.1% vs 23.5%, OR 0.92 (95% CI 0.72, 1.18), P = 0.52, I2 = 0%). However, this analysis demonstrated significant variation with time after intervention.

**Figure 3:**
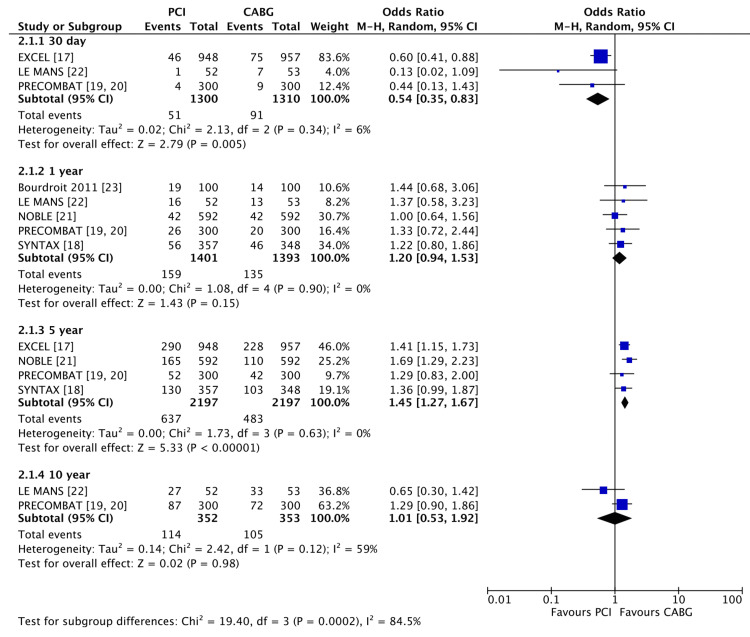
Forest plot of MACCE in PCI vs CABG at 30 days, 1 year, 5 years and 10 years Data shown compares MACCE in left main coronary artery disease when treated with PCI vs CABG [17-23]. PCI: percutaneous coronary intervention; CABG: coronary artery bypass grafting P < 0.05 was considered significant.

MACCE rates at 30 days were lower for PCI than CABG (Figure 3), as expected (3.9% vs 6.9%, OR 0.54 (95% CI 0.35, 0.83), P = 0.005, I2 = 6%). There was no difference between approaches at 1 year (11.3% vs 9.6%, OR 1.20 (95% CI 0.94, 1.53), P = 0.154, I2 = 0%) but at 5 years, PCI had higher rates of MACCE than CABG (28.9% vs 21.9%, OR 1.45 (955 CI 1.27, 1.67), P < 0.001, I2 = 0%). No difference was seen between PCI and CABG at 10 years in the two available studies (32.4% vs 29.7%, OR 1.01 (95% CI 0.53 vs 1.92), P = 0.98, I2 = 59%), but smaller numbers and potential biases may exist.

**Figure 4:**
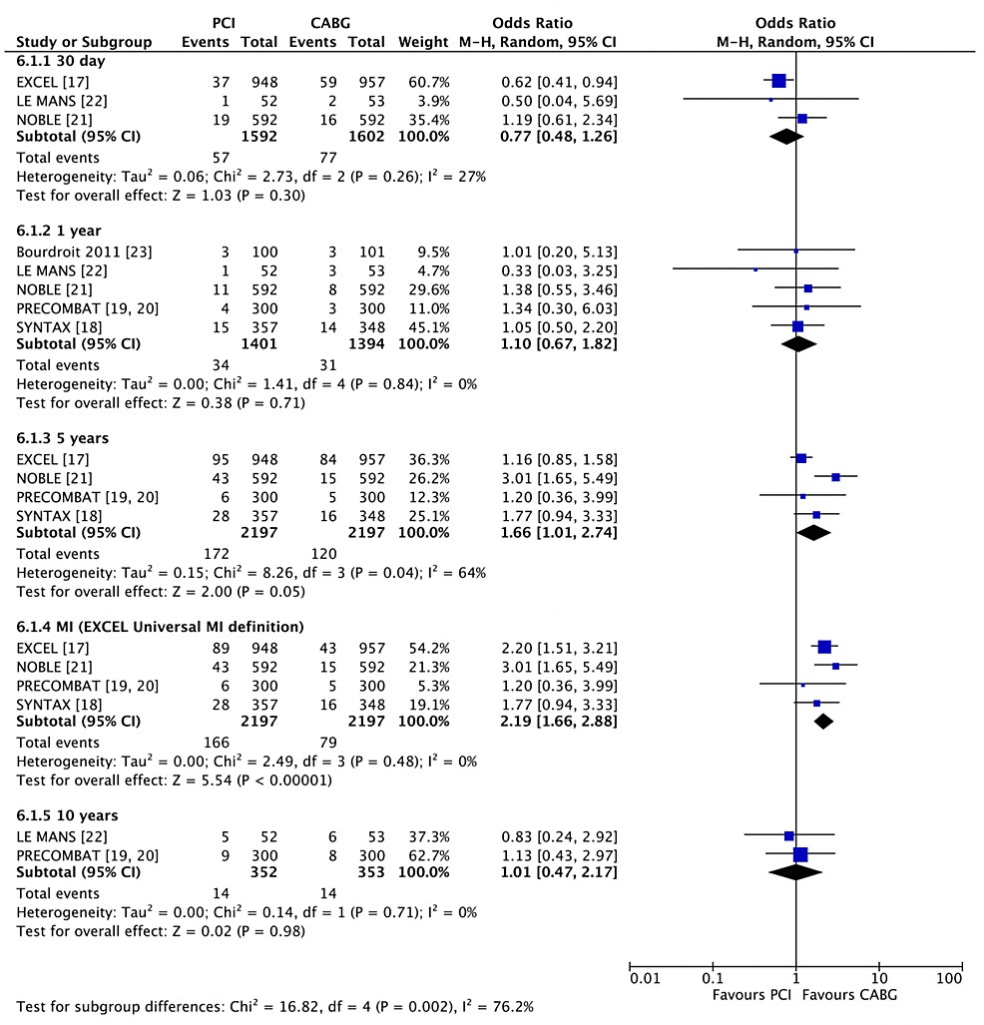
Forest plot of MI occurrence in PCI vs CABG at 30 days, 1 year, 5 years and 10 years Data shown compares MI occurrence in PCI vs CABG. Five-year data are plotted twice to incorporate both the originally reported EXCEL five-year follow-up MI data as well as the recently released five-year follow-up data generated using the third universal definition of MI [17-23]. PCI: percutaneous coronary intervention; CABG: coronary artery bypass grafting. P < 0.05 was considered significant.

Regarding the occurrence of MI (Figure 4), there was no significant difference between the PCI and CABG groups at 30 days (3.5% vs 4.8%, OR 0.77 (95% CI 0.48, 1.26), P = 0.30, I2 = 27%) and 1 year (2.4% vs 2.2%, OR 1.10 (95% CI 0.67, 1.82), P = 0.71, I2 = 0%). Using the original 5-year EXCEL trial definition, 5-year MI rates were higher for PCI than CABG (7.8% vs 5.4%, OR 1.66 (95% CI 1.01, 2.74), P = 0.05, I2 = 64%). When the re-analysed EXCEL data (using the Third Universal Definition of MI) was included instead, the 5-year data demonstrated a greater difference, with 7.5% MI rates in the PCI group compared to 3.6% in the CABG group (OR 2.19 (95% CI 1.66, 2.88), P < 0.0001, I2 = 0%).

**Figure 5:**
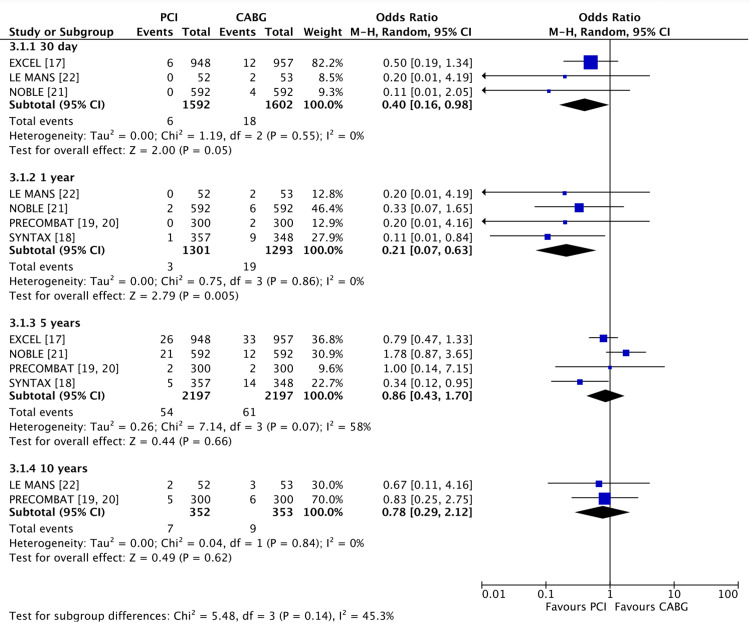
Forest plot of stroke occurrence in PCI vs CABG at 30 days, 1 year, 5 years and 10 years Data shown compares stroke incidence in left main coronary artery disease when treated with PCI vs CABG [17-23]. PCI: percutaneous coronary intervention; CABG: coronary artery bypass grafting. P < 0.05 was considered significant.

PCI showed lower rates of stroke (Figure 5) at 30 days (0.37% vs 1.1% for CABG, OR 0.40 (95% CI 0.61, 0.98), P = 0.05, I2 = 0%) and at 1 year (0.23% vs 1.4%, OR 0.21 (95% CI 0.07, 0.63), P = 0.005, I2 = 0%). However, there was no difference at five years (2.4% vs 2.7%, OR 0.86 (95% CI 0.43, 1.70), P = 0.66, I2 = 58%) or 10 years (1.9% vs 2.5%, OR 0.78 (95% CI 0.29, 2.12), P = 0.62, I2 = 0%).

**Figure 6:**
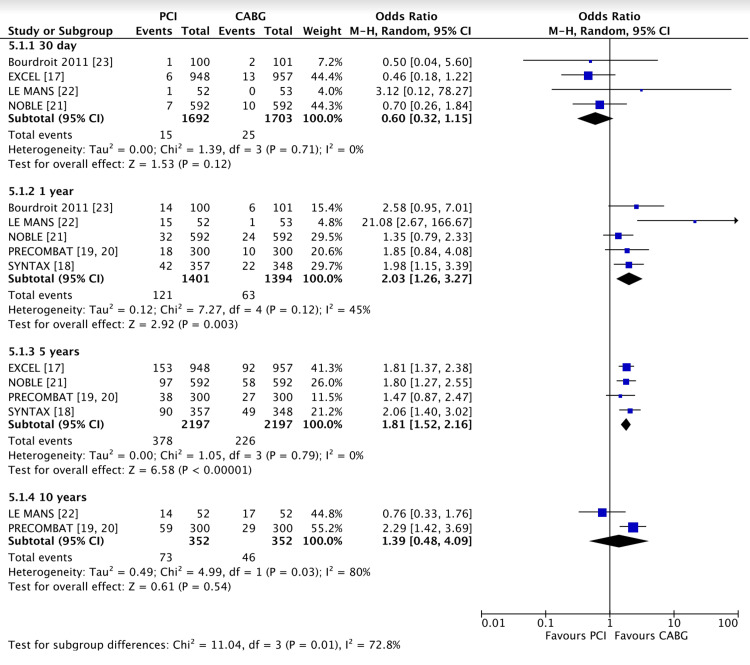
Forest plot of the revascularisation rate in PCI vs CABG at 30 days, 1 year, 5 years and 10 years Data shown compares the revascularisation rate in left main coronary artery disease when treated with PCI vs CABG [17-23]. PCI: percutaneous coronary intervention; CABG: coronary artery bypass grafting P < 0.05 was considered significant.

There was no significant difference in revascularisation rates (Figure 6) between PCI and CABG at 30 days (0.8% vs 1.4%, OR 0.60 (95% CI 0.32, 1.15), P = 0.12, I2 = 0%). However, subsequent time points demonstrated higher rates of revascularisation in the PCI group: 1 year (8.6% vs 4.5%, OR 2.03 (95% CI 1.26, 3.27), P = 0.003, I2 = 45%), and 5 years (17% vs 10%, OR 1.81 (95% CI 1.52, 2.16), P = 0.00001, I2 = 0%). Although no significant difference between revascularisation rates in PCI and CABG groups at 10 years (20% vs 13%, OR 1.39 (95% CI 0.48, 4.09), P = 0.54, I2 = 80%) was observed, the smaller numbers in the studies and significant potential biases make this data unreliable.

A summary of the assessments of the risk of bias in the studies is outlined in Table 3. Across all studies, there was a high risk of bias in the blinding of participants and personnel (performance bias). This is due largely to the nature of the interventions, as it is not possible to blind either the participants or the administrators to the intervention. The PRECOMBAT and LE MANS studies were deemed to have an unclear risk of (potentially severe) bias regarding incomplete data at the 10-year follow-up, as they were unable to obtain information from all non-deceased study participants (attrition bias) - less than 25 in each group were able to be contacted at 10 years. The EXCEL study and the PRECOMBAT study were determined to have a high risk of bias for selective reporting (reporting bias). The PRECOMBAT study did not report the rate of periprocedural MI, which may have influenced the analysis of MI occurrence between the two groups. The EXCEL study had initially reported rates of MI using a trial-specific algorithm and has since released data that classifies MI according to a more universally accepted standard. In the interest of transparency, both sets of data have been included in our analysis. All other sources of potential bias were deemed to be of low risk.

**Table 3:** Risk of bias assessment The risk of bias assessment was performed according to the Cochrane Guide of assessing the risk of bias in included studies.

Study	Sequence generation	Allocation concealment	Blinding of participants and personnel	Blinding of outcome assessment	Incomplete outcome data	Selective reporting	Comment
EXCEL [17]	Low	Low	High	Low	Low	High	Questions have arisen surrounding the reporting of data as being misleading or fraudulent. Also, participants and personnel could not be blinded due to the nature of the intervention
SYNTAX [18]	Low	Low	High	Low	Low	Low	Participants and personnel could not be blinded due to the nature of the intervention.
PRECOMBAT [19,20]	Low	Low	High	Low	Unclear	High	For the ten-year analysis, it states participants were lost to follow-up, and some data were inferred. Participants and personnel could not be blinded due to the nature of the intervention. Did not report periprocedural MI occurrence.
NOBLE [21]	Low	Low	High	Low	Low	Low	Participants and personnel could not be blinded due to the nature of the intervention.
LE MANS [22]	Low	Low	High	Low	Unclear	Low	For the ten-year analysis, it states participants were lost to follow-up, less than 25 in each group could be contacted at ten years. Participants and personnel could not be blinded due to the nature of the intervention.
Bourdroit 2011 [23]	Low	Low	High	Low	Low	Low	Participants and personnel could not be blinded due to the nature of the intervention.

Discussion

This systematic review is the first to include the five-year data from the large EXCEL trial, as well as the 10-year follow-up data from the Le MANS, PRECOMBAT, and SYNTAX trials [10,24-27]. This expanded data provides a comprehensive view of the long-term outcomes of both interventions in LMCA disease. Importantly, there was no difference in all-cause mortality at all time points. Revascularisation was more commonly required in the PCI group at one and five years, in line with previous studies of revascularisation in other coronary arteries, although the overall rates of revascularisation were low - 8.6% for PCI and 4.5% for CABG at five years. Rates of myocardial infarction were similar at one year, but at five years, there was a higher proportion of MI in the PCI group (7.5% versus 3.6% for CABG) when incorporating the updated MI definition in the EXCEL trial (OR 2.19). The rate of stroke was lower in the PCI group at 30 days and one year, but this difference disappeared by the 5- and 10-year follow-ups. Rates of MACCE were equivalent at one year but lower for CABG at five years, although this composite outcome was mainly driven by lower rates of revascularisation. The absence of a significant difference in all-cause mortality across all follow-up time points suggests that PCI is equivalent to CABG. The data showed good homogeneity at 30 days, 1 year, and 10 years, but the 5-year data had a relatively high heterogeneity score of 40%, partially attributed to a lower mortality in the CABG group at 5 years. However, this difference was believed to be driven by higher rates of malignancy and sepsis in the PCI group, which could not be directly attributed to the intervention and were coincidental.

Regarding myocardial infarction data at 30 days post-intervention, there was a non-significant trend favouring PCI over CABG, but the outcomes were quite heterogeneous due to the limited number of studies reporting data at 30 days. At 1-year and 10-years, no significant differences were observed between either intervention. Analysing the five-year follow-up data, both the originally reported EXCEL MI data and the recently released data using the Third Universal Definition of MI were included. The 5-year follow-up data that included the original EXCEL data significantly favoured CABG over PCI (OR 1.66, 1.01-2.74), and when the data was reanalysed using the Third Universal Definition of MI, the results favoured CABG even more (OR 2.19, 1.66-2.88). This suggests the original definition of MI used in the EXCEL trial may have overreported MI occurrence in the CABG group, with the total number of MI events nearly 50% lower following the switch to the Third Universal Definition of MI (n = 84 vs n = 43). Another notable finding was the significant effect of the NOBLE trial results on the five-year outcome data. While all the five-year follow-up data trended towards favouring CABG, the NOBLE data showed the strongest support for CABG over PCI (OR 3.01, 1.65-5.49). One reason may be the absence of periprocedural MI reporting in the NOBLE trial, a measure that was included in the other studies. This may explain why the NOBLE data strongly favours CABG, as periprocedural MI has been shown to occur more frequently in CABG when compared to PCI, potentially affecting outcome [28].

Revascularisation rates at 1 and 5-year follow-up both favoured CABG, with 10-year data also trending toward CABG (although with more limited data). The superiority of CABG at these time points is consistent with other studies of coronary revascularisation, where rates were lower for PCI compared to CABG. The use of the internal mammary artery (IMA) during CABG is known to confer particular benefits for reducing the future need for revascularisation, and this was the most frequently utilised graft in all trials except the SYNTAX trial [29]. Lower rates of stroke were observed in the PCI group at 30 days and 1-year follow-up, with all the included studies showing odds ratios that favoured PCI at both time points. The superiority of PCI was not maintained at the 5- and 10-year follow-ups; however, the odds ratios at each time point trended towards favouring PCI. This finding of non-inferiority of CABG at five years contrasts with the findings of two earlier systematic reviews [3,30]. A more recent study that included the same RCTs as this review, however, concurs with our observation [10].

Combining individual outcomes into a single outcome (MACCE) resulted in similar rates at one year, but by five years, CABG demonstrated lower MACCE rates. The 10-year follow-up data did not show any significant difference between PCI and CABG, but only two studies have reported 10-year MACCE data thus far. These results are like a recent systematic review which found no difference between PCI and CABG at one year [20]. It is important to note that the superiority of CABG at five years was mainly driven by the higher rate of MI and revascularisations in the PCI arm. Additionally, the composite endpoint of MACCE has limitations, as it fails to address differences in quality-of-life post-intervention or the degree of disability associated with the event. While two interventions may share the same MACCE score, one may be due to increased revascularisation attempts while the other is due to increased post-interventional MI or stroke. Furthermore, the lack of consistency in the MACCE definition adds more uncertainty to the use of the composite outcome, with the EXCEL trial not reporting revascularisation as a MACCE event. Individual outcomes often provide greater clarity.

The results of this review show that PCI and CABG are both viable management strategies for LMCA disease, with the potential for selecting the intervention based on specific treatment goals and future risk balancing. There was no difference in mortality, but lower rates of revascularisation and MI were observed in the CABG group at one and five years. The trade-off of faster recovery and less invasive intervention with PCI (compared to CABG) in exchange for an increased risk of re-intervention over five years may be suitable for some while others may opt for CABG. This may also be of interest from a resource allocation standpoint, as a decreased need for revascularisation would theoretically necessitate fewer follow-up procedures. The significant decrease in the occurrence of MI at 5 years in the CABG arm suggests it provides longer-term protection against infarction, in keeping with studies in other parts of the coronary arteries. Despite the superiority of CABG at certain time points for MI and revascularisation, this does not translate to decreased mortality, which may reflect that the MIs involved were relatively small (although this information was not included in the published data). Despite these findings, the review has some limitations. Only six studies met the criteria for inclusion in this analysis, spanning a 12-year period. The nature of PCI has evolved significantly since the late 2000s when the first studies were conducted, and differences in the post-intervention protocols may have influenced the outcomes, with variations in the antiplatelet regimens used across the two different treatments varying significantly. Other limitations include the lack of consistency in the definition of MI, varying follow-up times, the use of different drug-eluting stents across different trials, and the overall moderate number of participants across the six trials. Over 40% of the total subjects in this meta-analysis were in the EXCEL trial, so the conclusions are significantly affected by this data in the statistical analysis.

## Conclusions

In conclusion, PCI may be considered an acceptable alternative to CABG for revascularisation of the LMCA. The risks and benefits are, however, different, and patient preference should be considered where the outcomes are similar. Further studies that compare the complexity of the lesion with the outcome would be welcome, as currently, CABG remains the preferred treatment for more complex anatomical lesions, but modern PCI techniques can result in good outcomes in complex lesions.
